# Cannabigerol Effect on *Streptococcus mutans* Biofilms—A Computational Approach to Confocal Image Analysis

**DOI:** 10.3389/fmicb.2022.880993

**Published:** 2022-04-29

**Authors:** Muna Aqawi, Doron Steinberg, Osnat Feuerstein, Michael Friedman, Sarah Gingichashvili

**Affiliations:** ^1^The Biofilm Research Laboratory, The Faculty of Dental Medicine, Institute of Biomedical and Oral Research, The Hebrew University of Jerusalem, Jerusalem, Israel; ^2^The Institute of Drug Research, School of Pharmacy, The Hebrew University of Jerusalem, Jerusalem, Israel; ^3^Department of Prosthodontics, Hadassah Medical Center, Faculty of Dental Medicine, The Hebrew University of Jerusalem, Jerusalem, Israel

**Keywords:** biofilm, *Streptococcus mutans*, confocal image analysis, computational assay, cannabigerol

## Abstract

Biofilms are complex bacterial structures in which bacterial cells thrive as a community. Many bacterial species, including pathogens, form biofilms of high complexity and adaptability to a wide range of environmental conditions. One example of these is *Streptococcus mutans*, a gram-positive bacterium that has been associated with caries. Cannabigerol, a non-psychoactive cannabinoid, has been shown to affect *S. mutans* biofilms. In order to better characterize the effect of cannabigerol on biofilms of *S. mutans*, this paper provides a series of computational assays for biofilm analysis, applied on confocal images of *S. mutans* biofilms treated with cannabigerol. Confocal images are ubiquitous in biofilm analysis—they are often used to visualize the complex structure and molecular composition of biofilm macrocolonies. In this article, we demonstrate how confocal imaging data can be used to reveal more comprehensive insights into biofilm structure and measure specific anti-biofilm effects. This is accomplished by a series of computational assays, each focusing on a different aspect of biofilm structure.

## 1. Introduction

Biofilms in nature are formed by many species of microorganisms (Flemming and Wuertz, 2019). These biological structures, consisting of cells and rich extra-cellular matrix, adhere to a multitude of surfaces, both natural and synthetic (Flemming et al., 2016). Biofilm formations are characterized by increased structural complexity that provides a protected micro-environment for the bacteria as it limits diffusion of anti-microbial agents, allows for cellular differentiation and communication and facilitates adherence to the host surface. Moreover, the structure and macro-morphology of biofilms have been found to be highly dynamic with different environmental conditions resulting in different morphologies and growth kinetics (Gingichashvili et al., 2019). For this reason, there is a need to characterize the relative ‘structural robustness' of biofilms in their native, undisrupted state, as it is directly correlated to key properties of the biofilm and its potential susceptibility to antimicrobial agents.

There are over 700 different bacterial species in the oral microflora (Faran Ali and Tanwir, 2012), and among them the Streptococci are highly associated with dental caries (da Silva et al., 2008; Steinberg et al., 2008; Tabchoury et al., 2008). These species colonize a wide range of oral surfaces such as the teeth, tongue, and oral mucosa. In the context of the oral cavity, *Streptococcus mutans* is considered as a leading cariogenic bacterium (Assaf et al., 2015). It is a gram-positive facultatively anaerobic bacterium, whose presence within the dental biofilm greatly influences the oral microflora and facilitates development of a potentially cariogenic biofilm in the oral cavity (Farkash et al., 2019).

Recently, natural cannabis-derived extracts emerge as potential tools for prophylaxis and treatment of different diseases (Ekor, 2014). Despite the therapeutic benefits of tetrahydrocannabinol (THC), its psychoactive properties limit its potential applications. Consequently, recent research has focused on other non-psychoactive cannabinoids such as cannabigerol (CBG) (Turner et al., 2017). CBG is a precursor of △9-THC and CBD (Brierley et al., 2016). *In vitro* and *in vivo* studies suggest a therapeutic potential for CBG in the context of medical therapy (Ekor, 2014; Nachnani et al., 2021). We have previously demonstrated that CBG has an antibacterial effect. It antagonizes the QS-mediated processes in *Vibrio harveyi*, including bioluminescence, biofilm formation, and motility, at concentrations that do not affect planktonic growth (Aqawi et al., 2020). Additionally, it exhibited both bacteriostatic and bactericidal effects on *S. mutans* (Aqawi et al., 2021a,b).

Accurate characterization of the effects of an anti-biofilm agent, such as CBG, is crucial for efficacy assessment and comparison of different agents. Many traditional biofilm assays require a disruption step—whether in molecular-based methods such as PCR or by way of measuring robustness by what is often termed “erosion assays” (Klotz et al., 2019). In order to view undisrupted biofilms, confocal laser scanning microscopy is often utilized (Sommerfeld Ross et al., 2014; Schlafer and Meyer, 2017). Indeed, confocal images are ubiquitous in biofilm research where they are used to illustrate an undisrupted section of biofilms. Unlike traditional methods for biofilm analysis, they do not require “disruptive” sample preparation steps—for example, CBG effect on *S. mutans* biofilm was previously explored using traditional assays that required overnight drying, induced lysis and shaking (Aqawi et al., 2021b). In addition to representing biofilms in their more “native” state, confocal image files hold two exclusive types of data: first, in terms of pixels that are distributed in 3D which enable depth-wise analysis of all biofilm layers, top-to-bottom. This is particularly important for studies that evaluate diffusion ability of antimicrobial agents—agents that are able to affect deeper layers of the biofilm are likely to be more effective in biofilm inhibition. Secondly, each individual pixel within the biofilm can hold multi-channel data representing more than one biological marker at each pixel—for example SYTO 9, PI, and AlexaFluor647, whose signal intensity represents live cells, dead cells and extracellular polysaccharides (EPS), respectively. Co-localization analysis of the various signals allows for an inside look into the cellular relationships within the biofilm and the changes that occur as a result of antimicrobial application—e.g., how is EPS production affected as a function of changes in SYTO 9/PI signals (live/dead cells) at different zones within the biofilm?

3-D computational analysis of images is well-accepted in medicine; MRI, CT, US being the most well known examples. These analytical methods are used as important diagnostic tools. Unique image features can be utilized to expand our knowledge of microbial biofilms. These computational, 3-D, image analyses will allow us to characterize key properties of the biofilm and better understand the micro-environment of the biofilms.

In the literature, computational biofilm morphology studies have shed some light onto the importance of structure to overall biofilm robustness. For example, analysis of *Vibrio cholerae* biofilm expansion kinetics and wrinkle formation reveal multi-stage growth curves and varying patterns of nutrient absorption (Fei et al., 2020). Wrinkles formed by mature *Bacillus subtilis* biofilms were previously shown to differ in structure under varying nutrient availability (Gingichashvili et al., 2019), while quantitatively estimated porosity and pore connectivity reveal the potential diffusing ability of complex 3-D biofilms (Rosenthal et al., 2018). COMSTAT (Heydorn et al., 2000; Vorregaard, 2008) and BiofilmQ (Hartmann et al., 2021) are both examples of software platforms that were specifically designed for the analysis of biofilm images, including confocal images. COMSTAT provides a platform that can be used to extract a substantial number of quantitative parameters from confocal biofilm images including biomass and surface area, while BiofilmQ provides a comprehensive image analysis platform with automated image segmentation and data analysis.

In this article, we propose a series of computational assays that can be used on confocal laser scanning microscopy (CLSM) images of undisrupted bacterial biofilms—specifically, *S. mutans* biofilms, treated with CBG. These virtual assays offer a specific layer-based approach that focuses on a comparative analysis designed for experiments with multiple biofilm samples. Moreover, the methods described in this paper offer a standardization process for confocal biofilm images that gives way for a mathematically fair comparison of biofilm samples. Thus, these computational methods allow for a comparative and reproducible analysis of the effects an anti-biofilm agent has on biofilm structure.

## 2. Materials and Methods

### 2.1. Bacterial Growth and Biofilm Construction

Planktonic *S. mutans* (UA159) were grown overnight at 37°C in 95% air/5% *CO*_2_ in brain heart infusion broth (BHI, Difco Labs, Detroit, USA). For biofilm formation, the overnight culture (Optical density of ~1.0) was diluted 1:10 in BHI containing 2% sucrose (BHIS). Two hundred microliters of the bacterial culture in BHIS were seeded with varying added cannabigerol concentrations (0–5 μg/mL) or ethanol 0.05% in each well of a 96 flat-bottom-well microplate and incubated for 24 h at 37°C in 95% air/5% *CO*_2_. Cannabigerol (CBG, hemp isolate, 95% purity) was purchased from NC Labs (Prague, Czech Republic) and dissolved in ethanol at a concentration of 10 mg/mL.

### 2.2. Spinning Disk-Confocal Laser Scanning Microscopy Analysis (SD-CLSM)

During biofilm formation, the cultures were incubated in the presence of 1 μL of a 1 mM AlexaFluor647-conjugated Dextran solution (10,000 MW, Molecular Probes Inc., Eugene, OR, USA) that stains exopolysaccharides. After 24 h incubation with the different concentrations of CBG (2.5 and 5 μg/mL), the early-stage or “plaque” biofilms were washed twice with PBS and then stained with 50 μL of live/dead [SYTO 9/propidium iodide (PI)] BacLight fluorescent dye (Molecular Probes, Life Technologies, Carlsbad, California, USA) for 20 min in the dark at RT.

The biofilm mass of untreated and CBG-treated biofilms was studied by live/dead SYTO 9/PI staining together with EPS staining (Duanis-Assaf et al., 2020), a technique where live bacteria emit green fluorescence, dead bacteria emit red fluorescence and EPS stained with Alexa Fluor647-conjugated Dextran appears as blue fluorescence. Immediately after incubation, the stained biofilms were washed with PBS prior to visualization under a Nikon Spinning Disk confocal laser scanning microscope (SD-CLSM) (Nikon Corporation, Tokyo, Japan) connected to Yokogawa W1 Spinning Disk (Yokogawa Electric Corporation, Tokyo, Japan). A three-dimensional (3-D) image of the biofilms was constructed using MATLAB [MATLAB. version 9.8.0 (R2020a), The MathWorks, 2020, Natick, Massachusetts, United States] and Imaris software (Imaris. version 9.8.0. Oxford Instruments, 2021, Abingdon, United Kingdom). At least three random fields were selected from each sample and analyzed.

### 2.3. Depth-Wise Inhibition Analysis

Original SYTO 9/PI/AlexaFluor647-conjugated Dextran staining data was obtained from SD-CLSM images—MATLAB scripts were used to deconstruct each confocal image into a 3-D numerical matrix, with pixel values ranging from 0 to 1 in each one of the three RGB channels, representing red, blue, and green primary colors. Each sample was measured for the sum intensity and normalized standard deviation values at each image layer (in the Z-axis). If we denote each layer size in pixels as *W* in width and *H* in height, the sum pixel intensity of each layer is defined as $\overset{W}{\underset{x=1}{\sum }}\overset{H}{\underset{y=1}{\sum }}I\left(x,y\right)$ with *I*(*x, y*) representing signal intensity (numerical value between 0 and 1) at pixel location (*x, y*). Likewise, if we denote $\bar{I}$ as the mean value of pixels at the layer and standard deviation as $\sigma =\sqrt{\frac{\overset{W}{\underset{x=1}{\sum }}\overset{H}{\underset{y=1}{\sum }}\left(I\left(x,y\right)-\bar{I}\right)}{\left(W\times H\right)-1}}$, normalized standard deviation is defined as $\frac{\sigma }{\bar{I}}$. The “central layer” of biofilms was defined as the single biofilm layer (2D slice) where sum pixel intensity value was largest, compared to all other layers.

### 2.4. Porosity and Intra-biofilm Connectivity

In order to address both aspects of porosity and intra-biofilm connectivity, we utilize an algorithm that identifies pore spaces at the central biofilm layer—biofilms with larger pore sizes are better interconnected and thus more susceptible to diffusion. Porosity calculations were performed using an algorithmic image processing pipeline to detect pore regions (Rabbani and Salehi, 2017; Ezeakacha et al., 2018).

### 2.5. Signal Correlation

From a computational point of view, SD-CLSM images can be viewed as three-dimensional matrices of pixels, much like natural images are represented in digital form—each pixel is a three-valued vector in a three-dimensional space, representing the intensity of live (SYTO 9), dead (PI), and EPS (AlexaFluor647) signals emitted from that point. Each biofilm sample is therefore a collection of pixels, the number of which equals layer size (width × height), multiplied by the number of provided Z-stack layers in the confocal image. Correlation between different channel values (live/dead/EPS) and their distribution in space can provide more detailed insights regarding the effects of the treatment. For each sample category, we compute the pairwise correlation coefficient (Pearson's linear correlation coefficient) between vectors of different channel values—live, dead, and EPS. Changes in the correlation values as a result of CBG treatment of different concentrations are quantified using pairwise linear correlation coefficient, computed using MATLAB software.

### 2.6. Histogram-Based Quantification

**Figure 1:**
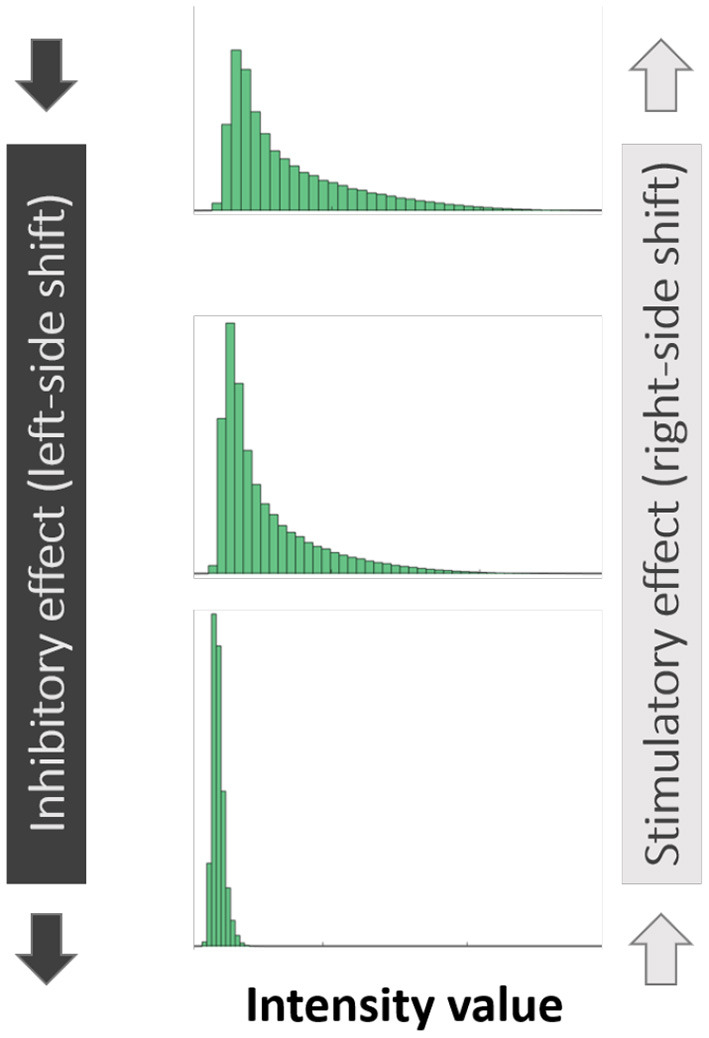
Illustration of “left-sided” and “right-sided” shifts in histograms of pixel intensity.

As is the case with image analysis, histograms provide a valuable view of the signals present in the image. Given a confocal image of any particular channel (live/dead/EPS), all pixel values in the 3D image (values in 0–1 range) can be visualized using a histogram which presents for each pixel value range, the number of pixels whose intensity value falls within that range. The ranges are determined by the number of “bins” specified—e.g., 10 bins would result in bins [0–0.1], [0.1–0.2] up to [0.9–1]. While a histogram view loses all spatial information, the changes that occur in the histograms of SD-CLSM images post treatment are a good visual representation of the overall anti-microbial effect, represented by shifts of the histograms. Given a SD-CLSM image, a vector of its original pixel values is generated for each of the three channels that represent live, dead and EPS signals. For each biofilm image a histogram is generated where for each pixel value range (“bin”), the number of pixels whose intensity value fits that bin is computed. Lower numbered bins represent pixels of low intensity values while higher numbered bins represent pixels of high intensity values. A “shift” of the histogram to the left is therefore indicative of a biofilm that has an overall reduced intensity value, while the opposite is true for a “right” shift (Figure 1). These shifts are quantified and the delta between them shows the shift of values between the sample groups in our data.

### 2.7. Statistical Analysis

The experiments were performed independently three times in triplicates. Between 8 and 9 images for each data set were obtained. Images were taken randomly to include areas at the middle and the edges of the biofilm. The data was analyzed statistically using Student's *t*-test in Microsoft Excel, with a *p*-value of <0.05 considered significant.

## 3. Results

### 3.1. Effect of CBG on *S. mutans* Biofilm (SD-CLSM)

**Figure 2:**
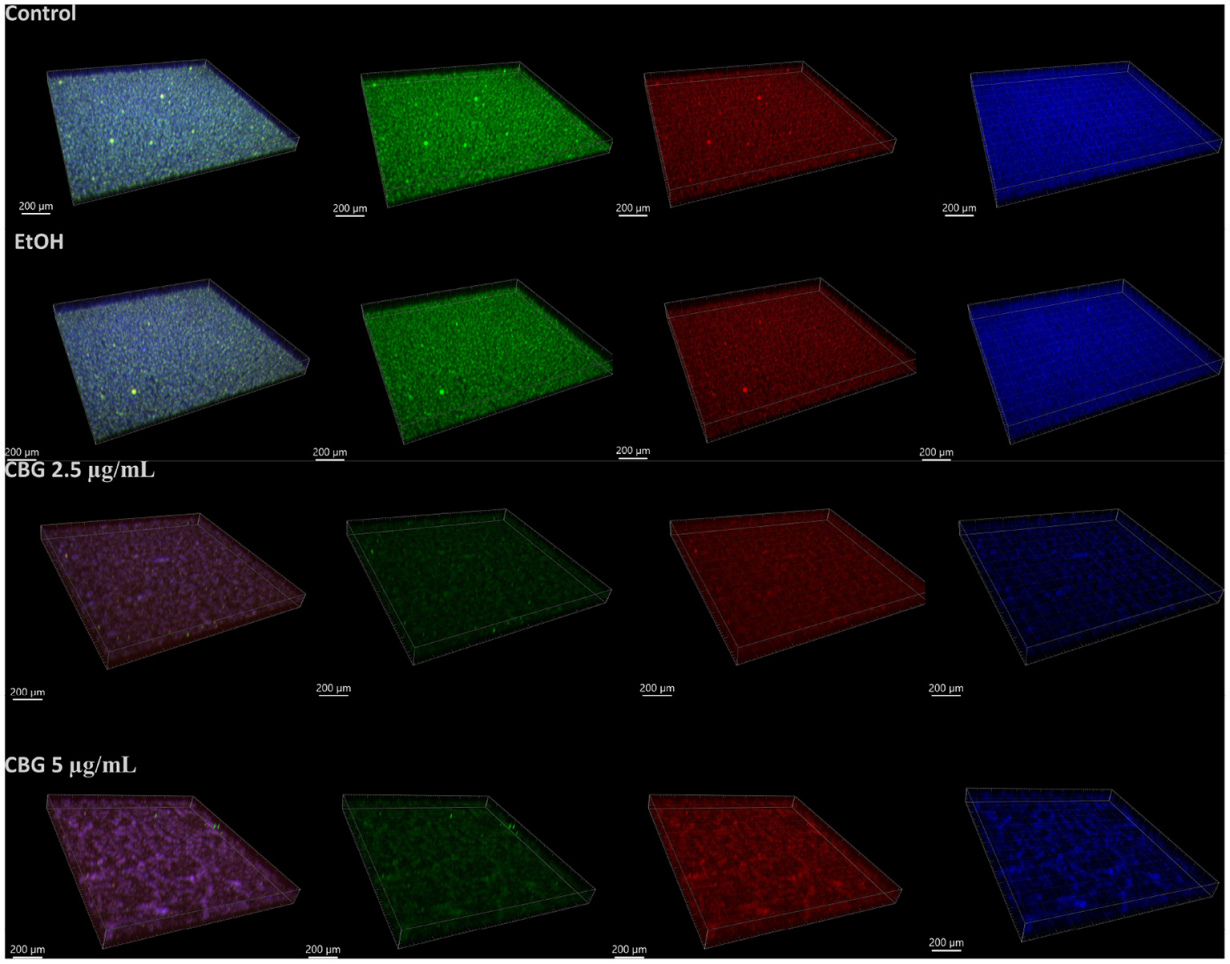
Spinning disk confocal laser scanning microscopy (SD-CLSM) images of SYTO 9 (green)/propidium iodide (PI, red)/AlexaFluor647-conjugated Dextran solution (blue). 3-D reconstruction of *S. mutans* biofilm samples—control and after treatment with EtOH (first and second rows) or CBG (third and fourth rows) for 24 h, as recorded by SD-CLSM and rendered using Imaris software. The four columns shown, left-to-right represent combined, SYTO-9, PI, AlexaFluor647 signals, respectively. A representative sample of each treatment is shown.

The biofilm mass of untreated and EtOH/CBG-treated biofilms was studied by live/dead (SYTO 9/PI) staining in combination with EPS staining (Figure 2), a technique where live bacteria emit green fluorescence (second column), dead bacteria emit red fluorescence (third column) and EPS, which is stained with Alexa Fluor647-conjugated Dextran, appears as blue fluorescence (fourth column). When visualizing the triple-stained images in Imaris software (Figure 2), the diminishing effect on the biofilm can be seen in the reduction of overall SYTO 9 signal intensity in CBG-treated macrocolonies, compared to untreated and EtOH-treated macrocolonies, representing a reduction in viable cells. Indeed, reduced signal density is observed in the blue channels, representing a reduction in the amount of EPS, when comparing CBG-treated to untreated/EtOH-treated macrocolonies. An increase in dead bacteria was observed in the samples treated with 5 μg/mL CBG (Figure 2).

### 3.2. Original SD-CLSM Data—Live (SYTO 9) Signal Intensity and Variation

**Figure 3:**
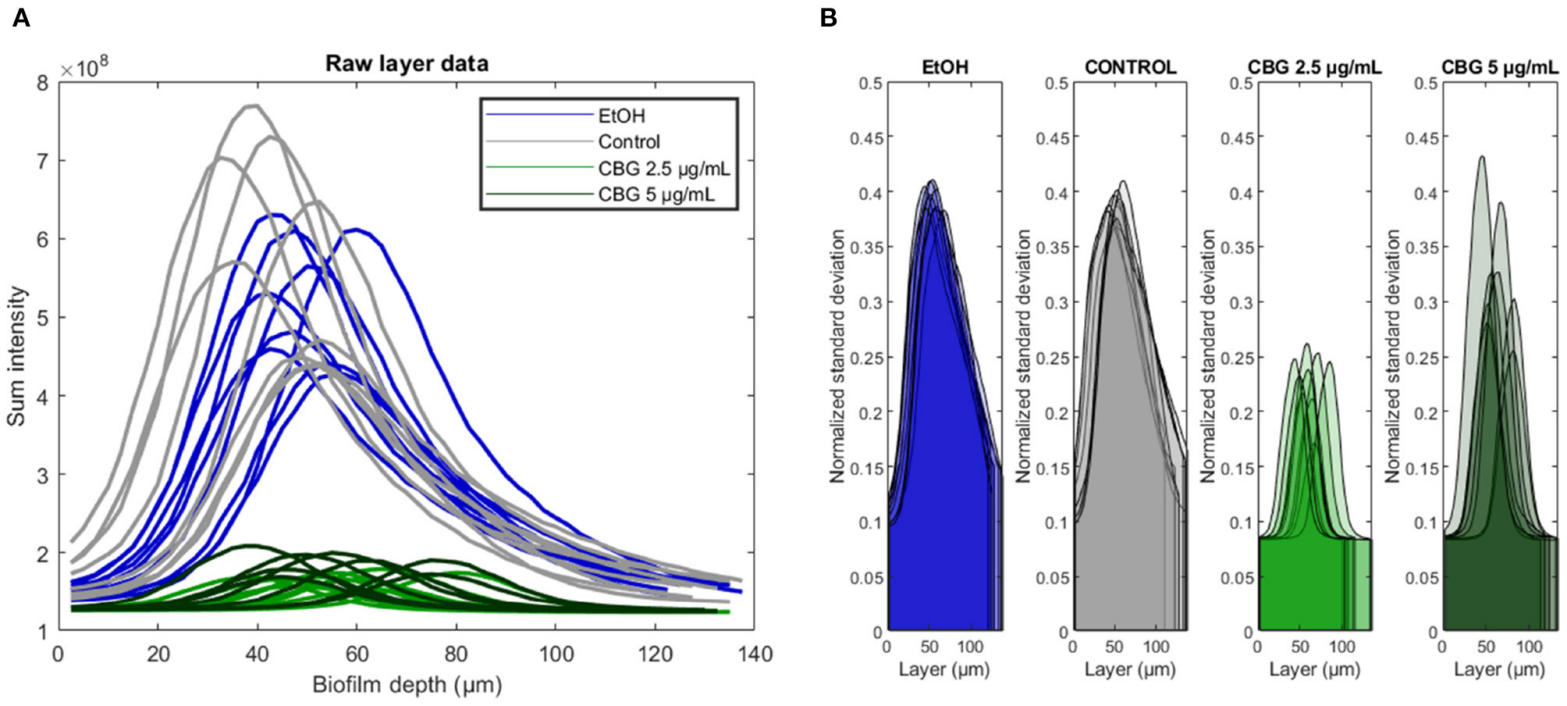
Original SYTO 9 values signal analysis. **(A)** Biofilms were analyzed depth-wise with the total SYTO 9 intensity shown for each biofilm layer. Central biofilm layer is defined as the layer at which the sum intensity was largest. **(B)** Depth-wise analysis of SYTO 9 signal normalized standard deviation across different biofilm layers.

For each biofilm sample, we compute a vector of sum pixel intensity values for each available layer i.e., specific biofilm depth. This analysis results in a vector of values, the length of which matches the number of Z-stack layers, as they appear in the original SD-CLSM images. Figure 3A demonstrates these vectors—it is important to note that all line plots appear to increase monotonically until they reach a “central” layer of maximal sum pixel intensity, after which they decrease monotonically. Figure 3A shows that the highest SYTO 9 signal was observed in the control group followed by EtOH. CBG treatment led to decrease in SYTO 9 signal intensity, hence less viable cells. It is noteworthy to mention that the CBG treatment did not show a dose-dependent effect as CBG at 2.5 and 5 μg/mL had similar effects. Figure 3B demonstrates that biofilm samples differ not only in the location (i.e., depth) and intensity of the central layer but also in the distribution of intensities found at its respective depth (measured by normalized standard deviation)—suggesting that some samples are more uniform at the central layer than others. In other words, cells at the central layer in control and EtOH-treated biofilms are located in a more varying environment, in terms of live cells' signal intensity, when compared to CBG-treated biofilms (Figure 3B).

**Figure 4:**
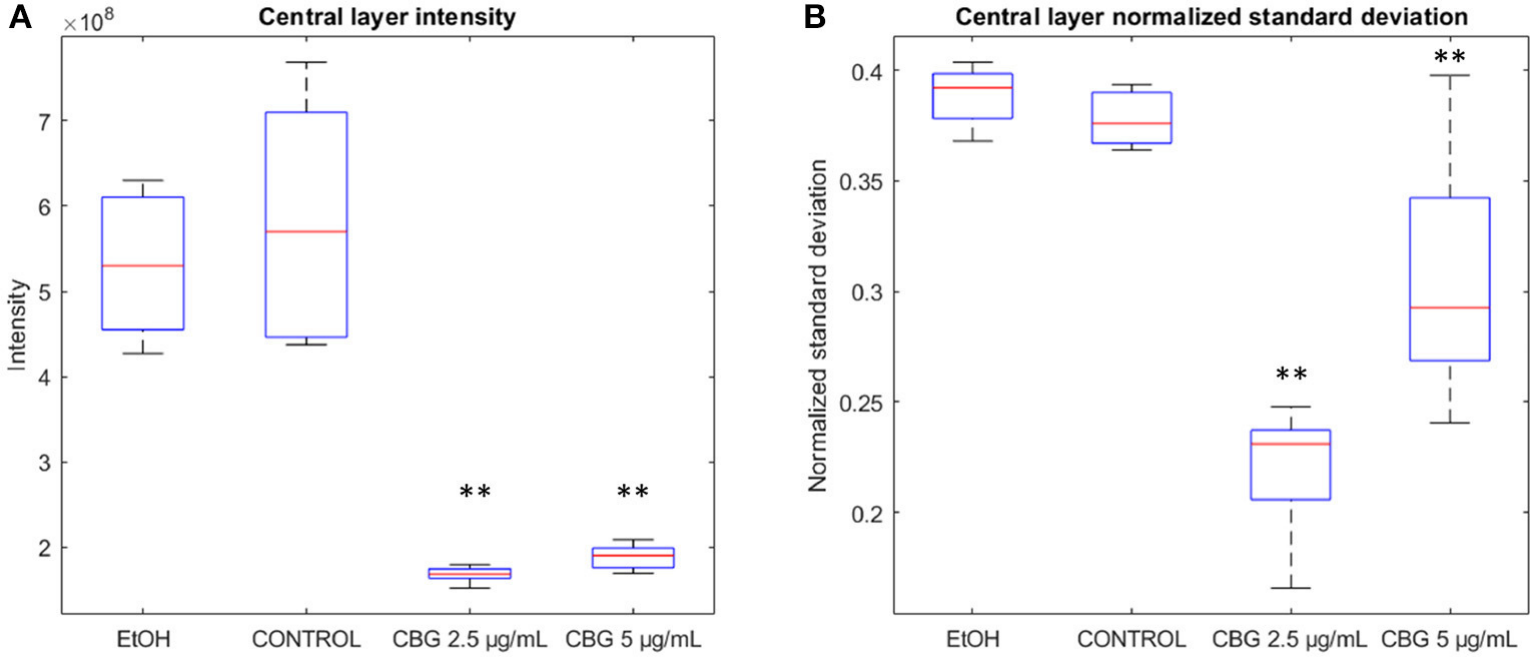
Basic features of the biofilm. **(A)** Central layer sum pixel live (SYTO 9) intensities. **(B)** Normalized standard deviation of pixel intensities at the central layer. Two-tailed *t*-test was performed. **p < 0.01.

Figure 4 illustrates differences in the characteristics of the central layer of biofilms—CBG-treated biofilms are characterized by a significant reduction in the intensity of the central layer, in addition to reduced normalized standard deviation. In terms of intensity, the amount of SYTO 9 signal, representative of live bacterial cells, in the central layer of CBG-treated biofilms is significantly reduced when compared to EtOH/untreated biofilms: when compared to control samples, EtOH, CBG 2.5 and 5 μg/mL samples exhibited reductions of 91.2, 28.9, and 32.5%, respectively. When assessing the live (SYTO 9) signal variation at the central layer, CBG-treated biofilms exhibited significantly lower normalized standard deviation values compared to EtOH-treated/control biofilms: when compared to control samples, EtOH, CBG 2.5, and 5 μg/mL samples exhibit values that are 102.5, 58.6, and 80.8%, respectively.

### 3.3. Realignment Around the Central Biofilm Layer

**Figure 5:**
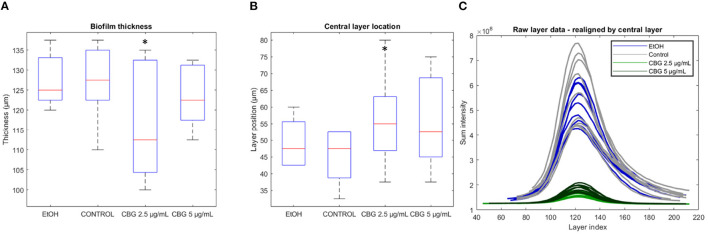
Biofilm thickness, central layer location, and realignment. **(A)** Biofilm sample thickness, calculated by the number of layers in SD-CLSM images multiplied by 2.5 μm per layer. **(B)** Depth in μm of the central layer. **(C)** Realigned layer-wise live (SYTO 9) signal intensity. Two-tailed *t*-test was performed. *p < 0.05.

The determination of where the biofilm “begins” and where it “ends” in terms of height, is done at the point of generating SD-CLSM images—often times the technician would scroll up and down to a height of a particularly low signal, setting that height as the cut-off. For example, in our dataset, each “scroll step” amounts to 2.5 μm. Such choice of cut-off is somewhat subjective and can result in artificially thick image samples. Figure 5A demonstrates differences in thickness values when those are computed directly from the number of Z-stack layers in original CLSM images. Average thickness values of control, EtOH, CBG 2.5 μg/mL and CBG 5 μg/mL were measured as 127.8, 127.5, 115.8, and 123.4 μm, respectively. Statistically significant differences were observed only in the 2.5 μg/mL CBG group. Figure 5B demonstrates the varying locations (i.e., depths) of the central layer in biofilm samples—average central layer location in control, EtOH, CBG 2.5 μg/mL and CBG 5 μg/mL was 45.3, 49.4, 56.1, and 55.6 μm, respectively. The central layer location was found to be statistically significant only in the 2.5 μg/mL CBG group, when compared to control. In order to overcome the subjective nature of determining the biofilm layer depths, we propose realigning the original intensity data around the central layer. In other words, the relative location of the “peak intensity layer” after realignment is to be matched to the same depth in μm. Such realignment allows for a direct comparison of different confocal samples, regardless of image acquisition parameters. The results of this process are visualized in Figure 5C, which demonstrates the realigned intensity data. Following data realignment, biofilms can be compared in terms of their thickness by comparison of layer ranges that symmetrically contain a fixed percentage of the area under the curve (AUC). Intensity data realignment is accomplished by an iterative algorithm which, given a target AUC value, determines the minimal layer range that contains at least the target AUC. In this analysis, biofilm samples that are more concentrated around the central layer will differ by exhibiting a narrow layer range. In other words, for a specific AUC percentage, the resulting layer range that will be sufficient to contain that AUC will be narrower than that of biofilms that are more dispersed around the central layer.

**Figure 6:**
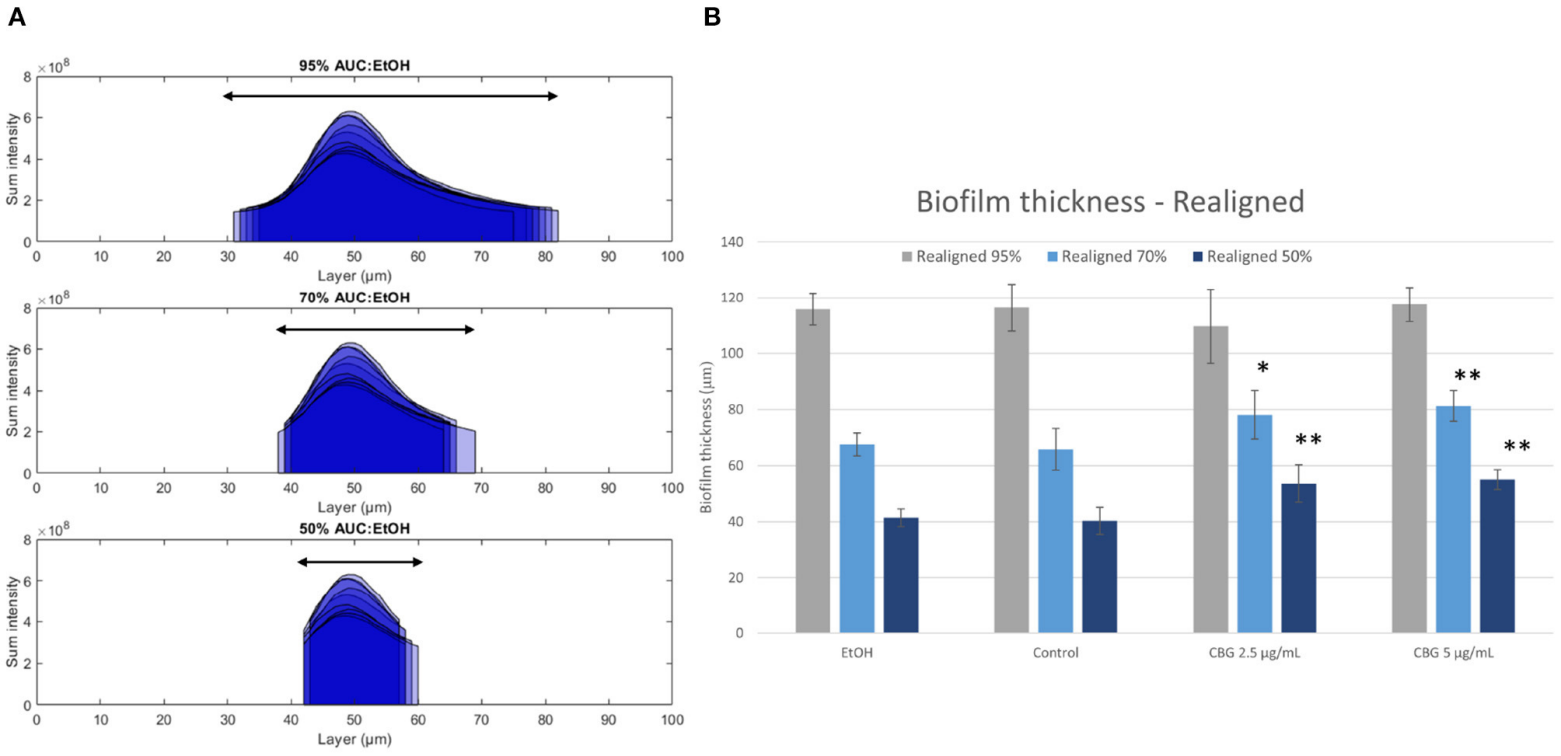
Realignment illustration. **(A)** Layer ranges that contain 95% (top row), 70% (middle row), and 50% (bottom row) of total AUC. **(B)** Thickness of minimal layer range that covers a fixed percentage of total AUC. Two-tailed *t*-test was performed. *p < 0.05, **p < 0.01.

Figure 6A illustrates the abovementioned realignment process by demonstrating values for 95% (top), 70% (middle), and 50% (bottom) AUC for EtOH-treated biofilms—black arrows illustrate the resulting layers' span. The biofilm thickness which spans the resulting values is calculated for all categories and is shown in Figure 6B. When comparing the thickness of CBG-treated biofilms to EtOH/untreated samples, no statistically significant differences in layer ranges are observed at 95%. However, when limiting the analysis to the layer range that contains 70 or 50% of the total AUC, CBG biofilms exhibit larger ranges than their EtOH/untreated counterparts. This phenomenon reveals that while EtOH/untreated biofilms are concentrated around their central layer, CBG biofilms are more loosely distributed around it—in other words, their height-distribution is wider than that of EtOH/untreated. Due to this wider distribution of CBG-treated samples, a wider range of layers is necessary in order to sufficiently contain a fixed percentage of AUC, compared to EtOH/untreated biofilms (Figure 6). This suggests that CBG biofilms are more dispersed around the central layer than their control and EtOH-treated counterparts.

### 3.4. Porosity and Intra-biofilm Connectivity

**Figure 7:**
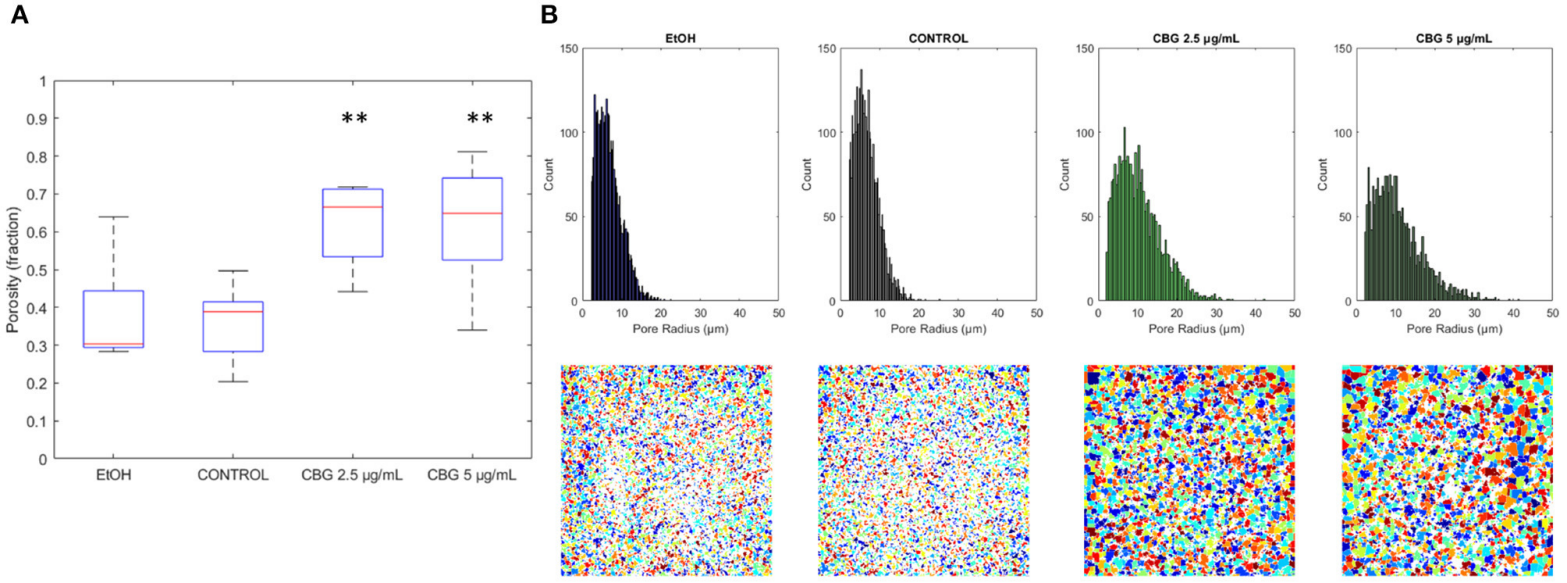
Central layer porosity. **(A)** Boxplot of porosity values at the central layer. **p < 0.01. **(B)** Pore size distribution is shown in the top row, with representative images of pore spaces at central layers of each category shown in the bottom row.

Figure 7A demonstrates the differences in overall porosity, represented by the fraction of pore space in the central layer of the biofilm—porous biofilms are not only less robust overall but also more susceptible to diffusion by anti-microbial agents. Average porosity values of control, EtOH, CBG 2.5 μg/mL and CBG 5 μg/mL were measured as 0.36, 0.38 (105.7% of control), 0.62 (170.4% of control), and 0.62 (171.4% of control), respectively. Both CBG concentrations differed from control in a statistically significant manner. Figure 7B further demonstrates the differences in pore size distribution as seen in histograms of pore radii at the central layer (top row). CBG-treated biofilms exhibit longer tails, indicative of presence of larger pore sizes. Indeed, CBG-treated biofilms' maximal pore size at the central layer was found to be 135.4% (at 2.5 μg/mL CBG) and 204.6% (at 5 μg/mL CBG) larger than that of the control. These differences can also be visualized, as seen in representative renderings of all detected continuous pore regions (bottom row).

### 3.5. Signal Correlation

**Figure 8:**
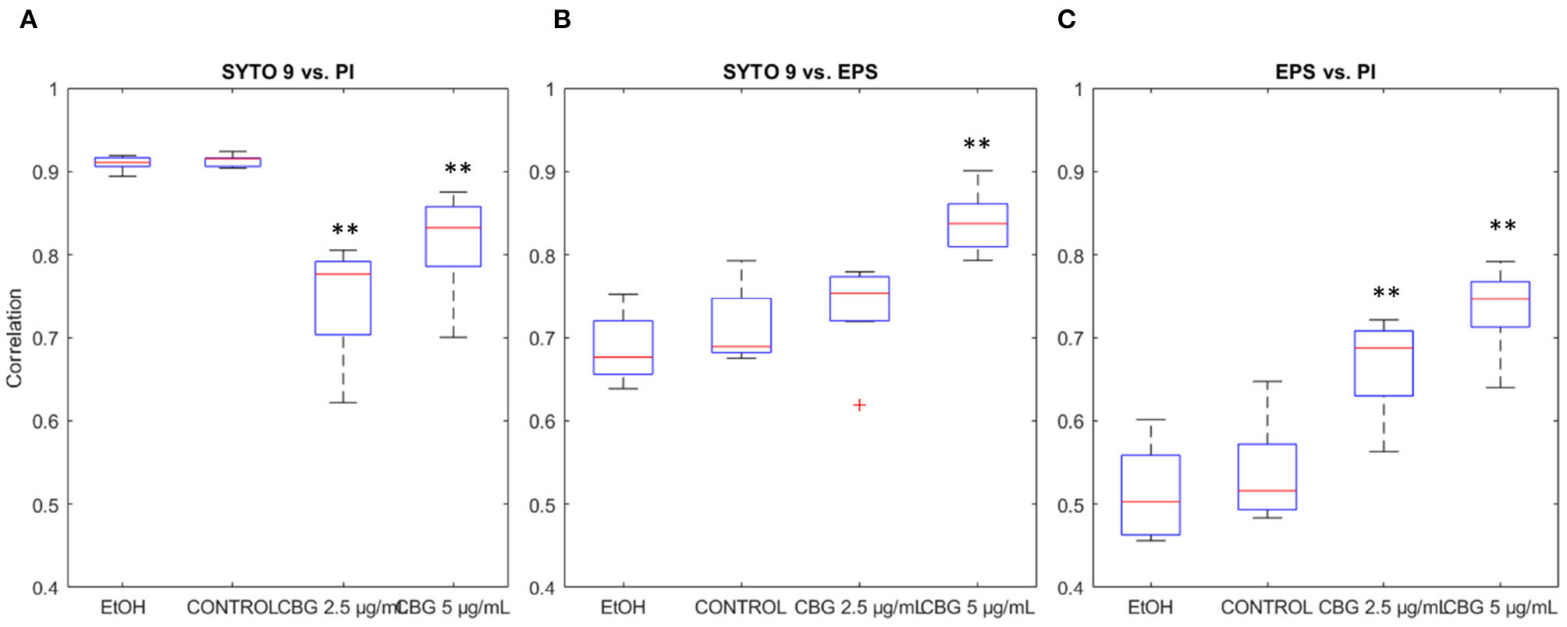
Signal correlation. **(A)** SYTO 9 vs. PI signal intensities correlation. **(B)** SYTO 9 vs. EPS signal intensities correlation. **(C)** SYTO 9 vs. PI signal intensities correlation. Two-tailed *t*-test was performed. **p < 0.01.

Each CLSM image in the input data is triple-stained for live cells (SYTO 9), dead cells (PI), and EPS (Alexa 647). Thus, each pixel is represented by a numerical vector that contains three values. Figure 8 demonstrates the differences in correlation that were found between each pair of signals. Statistically significant reduction (*p* < 0.05) in correlation was observed between SYTO 9 and PI signals between CBG-treated biofilms and EtOH/control biofilms (Figure 8A). Compared with control samples, correlation drops from 0.91 to 0.75 in CBG 2.5 μg/mL and 0.82 in CBG 5 μg/mL samples. When analyzing correlation between SYTO 9 and EPS signals, statistically significant increase in correlation was observed from 0.71 in control, to 0.84 in CBG 5 μg/mL samples. Statistically significant increases in correlation were also observed in EPS vs. PI intensity signals. Compared to control correlation of 0.53, CBG 2.5 μg/mL was increased to 0.67 and CBG 5 μg/mL was 0.74. In biological terms, there is more PI (dead cells) in the CBG-treated cells, whereas there is more live (SYTO 9) than EPS in the CBG-treated cells which means that there is a reduction in EPS in comparison to the number of cells. All in all, CBG resulted in more dead cells with less EPS production.

### 3.6. Reduction in EPS

**Figure 9:**
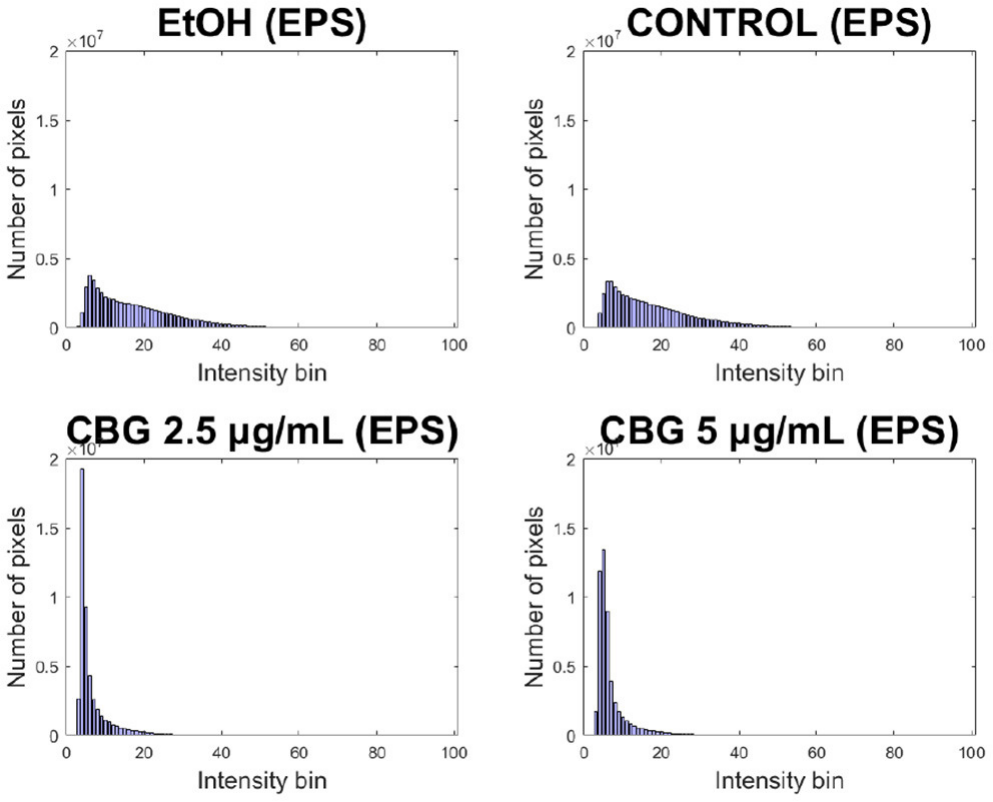
Left-sided shift in EPS production of CBG-treated macrocolonies.

Significant reduction in EPS signal of CBG-treated biofilm samples can be seen in Figure 9. Both CBG concentrations exhibit a “left-sided” shift, indicating the strength of overall reduction in EPS signal intensity. These results are similar to those presented by Aqawi et al. (2021b), both by high resolution scanning electron microscopy and Congo agar assay.

## 4. Discussion

Fluorescence confocal microscopy techniques offer a unique 3-D view into the internal structure of biofilms. In this work, we describe a series of quantitative computational methods to assess the anti-biofilm effect of CBG by using *S. mutans* as a model organism, as seen in SD-CLSM. Specifically, we utilize the SD-CLSM images to access the antibacterial effects of CBG, as these are reflected in 3-D, triple-stained (SYTO 9/PI/AlexaFluor647) biofilm images.

Previously, Aqawi et al. (2021b) demonstrated that CBG has an antibiofilm activity as it inhibits the formation of biofilms indirectly by acting as an anti-bacterial agent and directly by acting on metabolic pathways regulating biofilms. Additionally, it was shown to reduce expression of biofilm-regulating genes, EPS production, quorum sensing, and cause an increase in ROS production (Assaf et al., 2015). Here we show how we can utilize computational analysis to deduce additional biological effects. In this article, we present results of a computational analysis that reflect similar results of Aqawi et al. (2021b) using traditional methods.

### 4.1. Realignment of Data Around the Central Layer and Depth-Wise Analysis

Biofilm thickness is one of the parameters often used to deduce the anti-microbial effect of various agents (Suarez et al., 2019). This is due to the fact that thickness is directly correlated to susceptibility to anti-microbials as it is directly related to diffusion rate of each agent. However, biofilm thickness, if calculated directly by the number of Z-stack layers in CLSM images (Figure 5A), can be inaccurate as peripheral layers may not contain sufficient signal to be included as part of the biofilm structure. Therefore, we have proposed in this paper a realignment process (Figure 5C) that allows for a more accurate determination of biofilm thickness. By realigning biofilm layer data around the central layer of each sample, we are able to compare biofilms by thickness values that span a fixed percentage of the AUC. Figure 6B demonstrates that CBG-treated biofilms span wider layer ranges at 70 and 50%, when compared to untreated biofilms. For example, at 50% AUC coverage, CBG 2.5 μg/mL—53.6 μm, CBG 5 μg/mL—55.0 μm, with control and EtOH spanning 40.3 and 41.4 μm, respectively. This effect is not seen at 95% AUC, which demonstrates the differences in values distribution around the central layer—CBG-treated biofilms are more widely distributed than untreated/EtOH-treated biofilms. Taken together with the decreased live (SYTO 9) signal amplitude (Figure 5C), this signifies the differences in internal structure—CBG-treated biofilms are more “spread-out” than their untreated/EtOH treated counterparts.

### 4.2. Biofilm Porosity

Biofilms of equal thickness can vary in their internal structure—for example, porous biofilms that allow for easy passage of liquids in between their layers are more likely to be more susceptible to anti-microbial treatments than denser biofilms (Zhang and Bishop, 1994). Hence, a measurement of biofilm porosity can shed light on the potential susceptibility of biofilms to antimicrobial agents. The term “porosity” can be technically divided into two separate terms—the total non-cellular volume in the biofilm structure and a measure of internal connectivity i.e., how connected are the internal voids (or alternatively, bacterial cells). Figure 8 shows that CBG-treated biofilms exhibit not only a higher degree of porosity but contain larger pore sizes rendering CBG as a highly potent antimicrobial agent. Similar results were shown by Aqawi et al. (2021b), where high resolution scanning electron microscopy (HR-SEM) revealed comparable interspaces in CBG-treated biofilms, compared to untreated/EtOH-treated samples.

### 4.3. Staining Signals Correlation

Change in correlation between SYTO 9 and PI signals between CBG-treated biofilms and EtOH/untreated biofilms was found. This reduction in SYTO 9-PI correlation in the control/EtOH-treated biofilms, compared to CBG-treated biofilms, can be attributed to the nature of the SYTO 9 and PI staining. SYTO 9 binds DNA in both live and dead cells while PI enters only dead cells but also stains extracellular DNA (Rosenberg et al., 2019). Therefore, there is always a PI/SYTO 9 background in the image. However, according to Aqawi et al. (2021a), CBG increases the membrane permeability and PI uptake and hence the increase in the correlation is seen.

## 5. Conclusion

Quantitative and reproducible characterization of 3D, multi-channel fluorescent biofilm images is challenging in many cases. It is particularly difficult to perform accurate comparative analysis of such images, across different experiments. Our proposed model is an easily performed image-based analysis of *S. mutans* biofilms that enables us to characterize changes that take place during biofilm development under different treatments/growth conditions. Such computational analysis allows us to classify key biofilm properties and fairly compare between effects of different anti-biofilm agents. The process of biofilm formation can be numerically quantified from confocal laser microscopy images using objective parameters for biofilm image analysis, used to compare and monitor variations in biofilm structure.

## Data Availability Statement

The raw data supporting the conclusions of this article will be made available by the authors, without undue reservation.

## Author Contributions

MA, SG, and DS conceived the idea and wrote the paper. MA designed and performed the biological experiments. SG designed and performed the computational assays. MF and OF revised the manuscript critically and provided academic guidance. All authors have read and agreed to the published version of the manuscript.

## Funding

This research was partially supported by the STEP Graduate Training Program (STEP-GTP) and the Dr. Izador I. Cabakoff Research Endowment Fund.

## Conflict of Interest

The authors declare that the research was conducted in the absence of any commercial or financial relationships that could be construed as a potential conflict of interest.

## Publisher's Note

All claims expressed in this article are solely those of the authors and do not necessarily represent those of their affiliated organizations, or those of the publisher, the editors and the reviewers. Any product that may be evaluated in this article, or claim that may be made by its manufacturer, is not guaranteed or endorsed by the publisher.
